# Critical risk analysis of metals toxicity in wastewater irrigated soil and crops: a study of a semi-arid developing region

**DOI:** 10.1038/s41598-020-69815-0

**Published:** 2020-07-30

**Authors:** Yusra Mahfooz, Abdullah Yasar, Liu Guijian, Qamer Ul Islam, Amtul Bari Tabinda Akhtar, Rizwan Rasheed, Samina Irshad, Urooj Naeem

**Affiliations:** 1 Sustainable Development Study Centre, Government College University, Lahore, 54000 Punjab Pakistan; 2 Chinese Academy of Science (CAS)-Key Laboratory of Crust-Mantle Materials and the Environments, School of Earth and Space Sciences, University of Science and Technology of China, Hefei, 230026 Anhui People’s Republic of China; 3 District Officer Planning, City District Government Gujranwala, Lahore, Pakistan; 4 Department of Architecture and Town Planning, University of Engineering and Technology, Lahore, Pakistan; 5 Department of Architecture and Built Environment, University of Nottingham, Nottingham, NG7 2RD UK

**Keywords:** Environmental monitoring, Environmental impact

## Abstract

Toxic elemental exposure through consumption of contaminated crops is becoming a serious concern for human health. Present study is based on the environment and health risk assessment of wastewater irrigated soil and crops in a semi-arid region Faisalabad, Pakistan. The concentrations of potentially toxic elements (Cu, Cr, Mn, Fe, Pb, Zn, Ni) were analysed by atomic absorption spectrometer in five different crops (Corn, rice, wheat, sugarcane and millet), while, their topsoil’s and multi targeted risks analysis were assessed. Results showed, the mean values of Pb and Zn were higher in crop than Food and Agriculture Organization guidelines for food additives and contaminants. A strong positive correlation was found among wastewater and crop’s toxic metals (r^2^ values in Cu, Zn, Pb, Ni and Cr were 0.913, 0.804, 0.752, 0.694, 0.587 respectively). Whereas, a strong correlation was also found among soil and wastewater lead (r^2^ = 0.639). The calculations of Nemerow Integrated Pollution Index (NIPI) showed the soil samples maximum pollution limit (NIPI > 3) and Potential Ecological Risk Index (PERI) was found to be higher than maximum limit (PERI > 600) for all samples. While, for non-carcinogenic risk, Hazard Index (HI) values in adult were near threshold (HI > 1) for all crop samples. In children, the HI values for Corn, Rice and Wheat were above threshold limit and for Sugarcane and Millet, these were near to threshold. Cancer risk values for Cr found higher than safe limit (1 × 10^–6^) in adult and children for crop samples. Crop irrigation by wastewater irrigation is a prominent alternative option for water scarce countries, however prior testing and treatment of such wastewater streams must be employed to minimize the adverse impacts on human health and environment.

## Introduction

In recent era, the food safety and security has become one of the most significant problems towards human health^1^. Resources of fresh water are also being depleted and becoming scarce. As such it is evidently reported that by 2025, the 2/3 of world inhabitants might be suffering from water shortages^2,3^. The declining resources of freshwater and growing global population (i.e. about 9.2 billion by 2050) are posing considerable challenges for researching about the sustainable alternative options^4,5^. Due to climate change and water scarcities the farmers are now practicing wastewater irrigation in numerous countries. Whereas, around 20 million hectares in 50 countries are being irrigated by the metropolitan wastewater and has been accounted for an overall production of 40% of food. However, in the developing, where there are weaker policy and regulatory frameworks, such practices are often counterproductive and poses ecological and health risks. Pakistan is an agricultural country and most of its cultivational activities are accumulated within semi-arid and the arid climatic regions^6^. Whereas, about 30% of the wastewater is being used for the irrigation of about 32,500 ha land and about 64% of it is being disposed into rivers without any treatment. Wastewater irrigation practices are attractive for poor farmers due to a number of reasons like additional agricultural productivity and decreased cost of production^7^. In Pakistan, wastewater has been frequently (26%) used in urban and peri-urban regions that are deprived of any clean of treated water sources and also the untreated water is available at no cost hence it lowers the price of crop production by up to 60% i.e. in terms of fertilizers and pesticides etc.^8,9^. However, now with rising developments, rising health risks and concerns among societies and legislators are also enforcing pressures to minimise the anthropogenic activities and improve the level of social and public heath caused due to deterioration and toxicity of water and food supplies^10,11^. Examples of toxic impacts being posed by heavy metal contamination include the Minimiata and Itai-Itai diseases that had badly impacted the human health and environment^12^. Metals and metalloids that are existent in soils from natural fonts^12^ though, the maximum usage of agrochemicals has considerably enhanced metal impurity in agricultural soils^13^. Whereas, unplanned and long-term irrigation via unsafe water not only decreases the soil bearing and absorbing capacity against heavy metals, but also these can then infiltrate and accumulate in the groundwater and surface water resources, from where they can be ingested by the plants and crops. Such heavy metals do present a different behaviour than other plant contaminants, as these are non-degradable in nature, which make them bio-accumulative i.e. via food chains, hence rendering them as a critical threat towards human health^14–16^.

A lot of studies have deliberated that how toxic metals travel and enter in to human body via various routes of food chain and poses multiple detrimental impacts to human health^16–23^. Certainly, heavy metals are classified as critical toxicants of human food chain and are responsible for adverse health issues, ultimately causing minor health disorders to greater diseases such as cancer.

Agricultural soil is the main source and route of metals and metalloids travel in to the eatable parts of the plants and crops like root stem and leaves^24,25^. Although numerous studies have focused and analysed the source identification of such heavy metals however, there are multiple knowledge gaps where targeted research was needed. Especially the risk assessment for metal revelation via dietetic consumptions had been being lacking^26^ in many qualitative and quantitative aspects and also in-terms of addressing all major regions of Pakistan^27–30^. Therefore, present study is based on multi-variate pollution assessment, metals exposure and their health risk assessment towards the local inhabitants of semi-arid region in Pakistan. By wastewater irrigated soil and crops, so to provide a more holistic analysis to fill the existing data and information gaps.

## Methodology

### Description of study site

The Faisalabad city is located between Chenab and Ravi rivers known as lower Rachna Doab. It is second largest city of Punjab province in Pakistan. This city site is located slightly higher than the surrounding areas and has a mild slope from Northeast to Southwest. The topography of the area is generally flat with few hills. The climate of site is generally classified as a semi-arid (hot desert) with an average annual rainfall of 375 mm. The study was conducted around Chokera wastewater treatment plant in a semi-urban area of Faisalabad (31° 27′ 32″ North and 73° 0′ 20″ East), Pakistan. The wastewater treatment plant (oxidation ponds) was established in 1998, to meet the environmental obligations under Pakistan Environmental Protection Act 1997 and later the Punjab Environmental Quality Standards (PEQs)^31^. Whereas, wastewater is used round the year-round for irrigating the crop fields of 45 km^2^ in this area close to wastewater treatment plant. While, more than 200 farmers are working in close proximities during following cropping seasons of the year.

- Rice: November to January.
- Maize: spring and autumn, mid-Dec to mid-march and mid-May to august.
- Sugarcane: Rabi or fall sowing in September–November and 9spring sowing in February–March.

### Sample collection and preservation

**Figure 1:**
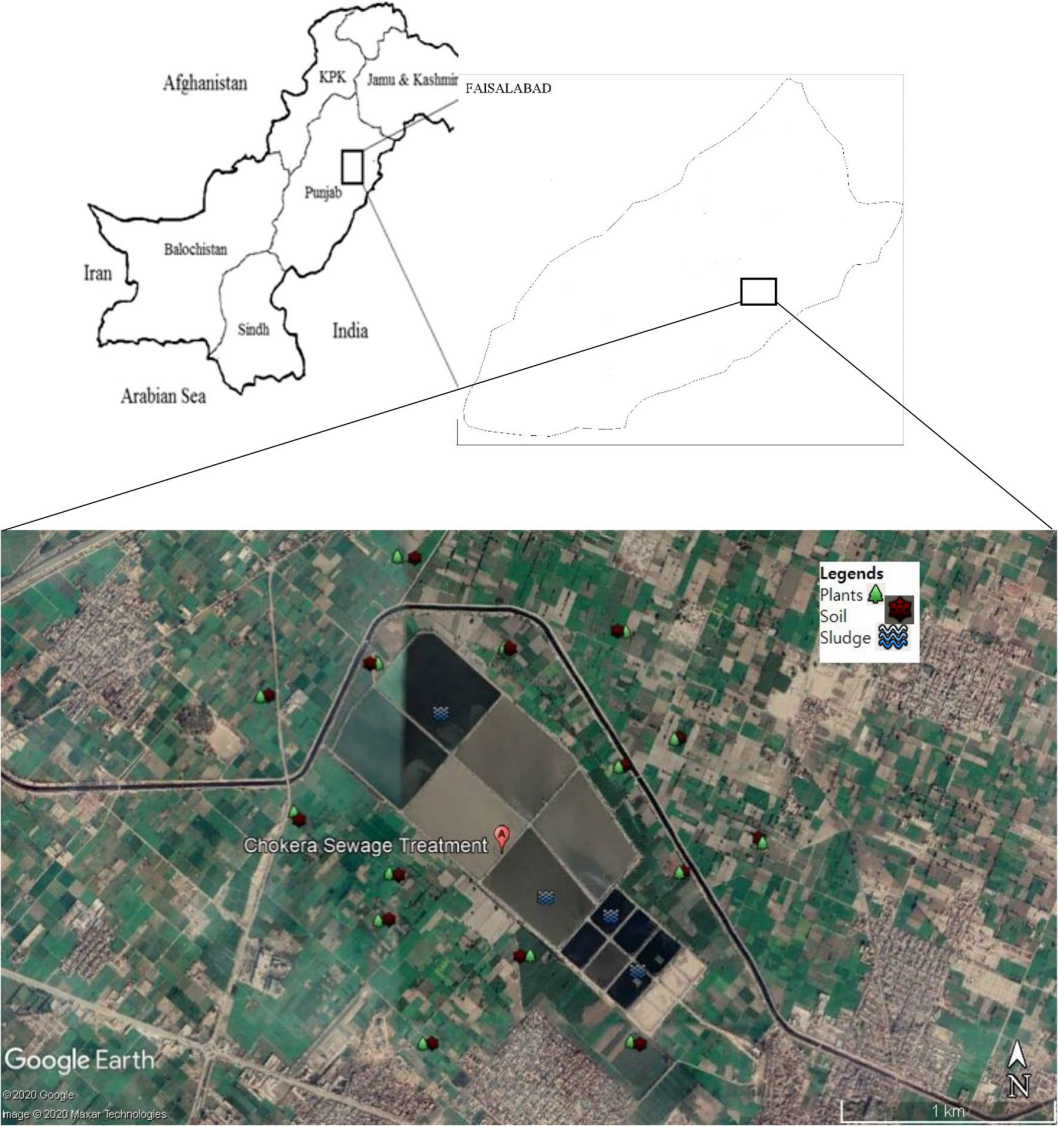
Study area map showing sampling locations (Satellite image created by using Google Earth Pro version 7.3.0.3832 https://www.neowin.net/news/google-earth-pro-7303832/).

Samples of five different crop field during their respective growing seasons with four replicates (combined to make it composite) of each field including wheat, rice, maize, sugarcane and corn with their surrounding soil (5–20 cm depth) samples (n = 20) were collected around the wastewater treatment plant which were being irrigated with untreated wastewater (Fig. 1). The each filed covered one-acre area approximately. The height of each plant was measured with scale at the time of sampling in the crop field, and their edible parts were collected. Wastewater samples (n = 52) were also collected from at various sites of Paharang and Mudhuana drains, which have been used for irrigation purpose. Sample were stored in plastic bottles and then transported to the laboratory at GC University, Lahore. Later the soil samples were oven dried (at 105 °C for 24 h), grinded and sieved (2 mm) and stored at 4 °C for further analysis. Crop samples were washed with tab water before rising with distilled water to removed dust. These were then dried in oven at 65 °C for 48 h, grinded and stored at room temperature.

### Analytical methods for soil and wastewater characterization

Soil texture was measured by using hydrometric method^26^ where, a sample has been converted to aggregate by treating it with sodium hexametaphosphate and then the organic matter becomes suspended in to solution. Later the density of soil was determined with hydrometer in g/L after the simultaneous settling of sand and silt. The density and temperatures of dispersing solution were also adjusted^32^. EC and pH were measured by means of a soil saturated extract by using EC digital meter (EC 300, YSI Company) and pH digital meter (pH 100, YSI company). Whereas, the CEC was measured by ammonium acetate extraction method and calculated by using following formula: CEC (cmol_c_/kg) = (NH_4_-N in extract—NH_4_-N in blank) / 18. In wastewater samples, the TDS and pH were measured by portable meters (EC 300, YSI Company and pH 100, YSI company respectively). While, the suspended solids (SS) were measured by calculating initial and final weight of dried filter paper. Moreover, the biochemical oxygen demand (BOD) and chemical oxygen demand (COD) were measured through Dissolved Oxygen Dilution Method within a BOD incubator for 5 days and Dichromate Reflux Method by using COD vials respectively. All methods were adopted from standards method for water and wastewater analysis^33^.

### Potentially toxic metal analysis

Samples were washed and oven dried. All the glassware, prior to use was washed with distilled water, rinsed with 10% nitric acid and dried. 1 g each for crop, sludge & soil samples were digested with the 15 mL mixture of H_2_SO_4_ and HClO_4_ and HNO_3_ (1:1:5) on hot plate until fumes appeared. Filtrates were cooled and filtered. Volume of digested sample was raised up to 50 ml by deionized water and then stored in refrigerator at 4 °C until metal analysis^34^. Metal (Cu, Cr, Fe, Ni, Mn, Zn and Pb) concentrations were evaluated by using Atomic Absorption Spectrophotometer (AAS Thermo Fisher Scientific iCE 3,000 series) at Government College University Lahore.

### Metal pollution indexes

Various pollution indices were calculated for determination of pollution levels in the soil and crop samples, whereas Nemerow integrated Pollution Index (NIPI) was used to quantify the said ecological pollution levels. Table 1 is depicting further classification of NIPI^35^ and PI under different categories^36^. While, the PI (Pollution Index) was calculated by using background values^37,38^. Potential Ecological Risk Index (PERI) was calculated according to Solomon et al.^39^ i.e. by the multiplication of C_s_^i^ (metal concentration) with C_n_^i^ (background value)^40^ and toxic response factor (T_r_^i^) values for Cu, Pb, Ni, Cr, Zn, Mn was taken as 5, 5, 5, 2, 1, 1^41–44^. The potential ecological risk factor of single metal (E_r_^i^) and potential ecological risk index are as categorized in Table S1^35^. The Bioconcentration Factor (BCF) is defined as translocation capacity of elements from soil to crop/plants. Here, BCFs of metals have also been evaluated^30^. Whereas the daily intake exposure (DIE) of metals was calculated according to method described by Yousaf et al.^26^ and Abbasi et al.^45^.

**Table 1:** Average concentrations (mg/L) of wastewater.

Sr. #	Parameters	Mean ± SD	Range	PEQs^a^ (2016)	FAO standards for irrigation
1	pH	9.155 ± 1.5	7.4–13.5	6–9	6.5–8
2	SS	247.3 ± 95.3	140–516	200	
3	TDS	4,007.15 ± 1,468	1,337–7,520	3,500	< 450
4	BOD	538.93 ± 589	63–4,061	80	200
5	COD	1,815.55 ± 2024	206–13,394	150	
6	Coliform	8,040.5 ± 36	3,600–14,400	–	< 200 per 100 ml
7	Arsenic	2.75 ± 1.8	0.11–4.34	1.0	0.10
8	Cadmium	0.95 ± 0.2	0.04–2.42	0.1	0.01
9	Chromium	1.685 ± 0.4	0.11–2.76	1.0	0.10
10	Copper	5.29 ± 4.8	0.06–9.34	1.0	0.20
11	Lead	1.175 ± 1.2	0.12–4.56	0.5	5.0
12	Nickel	6.7 ± 4	1.45–12.2	1.0	0.20
13	Zinc	13.32 ± 3.4	0.97–23.4	5.0	2.0

^a^Pakistan Environmental Quality Standards, number of samples = 52.

### Health risk index (HRI)

Three metal exposure routes in human have been enlisted including intake, inhalation and dermal but dietary exposure through food consumption is a considerable pathway^34^. Non-carcinogenic and carcinogenic risks are determined on metal consumption via crop intake. Whereas, the health risk index (HRI) in human via consumption of wastewater irrigated crops has also been calculated. RfD is a reference dose of metals including Cu (0.04), Zn (0.3), Mn (0.033), Cr (0.035), Pb (0.004) and Ni (0.02) as described by USEPA^46^. Non-carcinogenic risk is evaluated in-terms of a carcinogen exposure to any individual which increase the likelihood to develop a cancer in a lifetime, that is determined by calculating targeted hazard quotient (THQ) and hazard index (HI)^47^. While, if THQ > 1 the non-carcinogenic effect is likely to occur. The hazard Index (HI) is a total hazard quotient, where, HI < 1 means that an adverse impact would be unlikely to occur in the exposed population^26,35,46^.

Carcinogenic risk (CR) states the incremental likelihood of occurrence of any kind of cancer into a person during his/her lifetime. Whereas, SF is cancer slop factor for each element^48^. Hence the CR values have been evaluated only for Cr and Pb as their availability was only limited to these slope factors^49^.

### Statistical techniques

The statistical analysis includes the analysis of correlations that was performed by using MS Excel and Origin version 9.0.

## Results and discussion

### Potentially toxic elements fate in crop and soil samples

Wastewater characterization was carried out to determine the effects of their physio-chemical parameters on soil and crops (Table 1). All parameters of wastewater are exceeding the limits as indicated by Food and Agriculture Organization (FAO)^50^ i.e. for the reuse of wastewater in irrigation/cultivation.

Table 2 showed the results of mean values of different toxic elements in crop samples. Wastewater irrigated food crops values were compared with (FAO)/(WHO) 2001^51^ guideline cited by Yousaf et al. and Khan et al.^26,27^. A brief detail of crops is given in Table S2. The values of zinc were higher than the permissible limit in sugarcane and millet samples while lead found higher in all crop samples. Table 3 presented the average concentration of cultivated soil (mg/kg) samples and their values were compared with European Union standards^52^. Only zinc was found higher in corn, rice, sugarcane and wheat than their permissible limits other metals were within the standards. The order of higher metal concentrations in soil was as follow: Fe > Zn > Mn > Ni > Cu > Pb > Cr. Table S3 showed the properties of base soil of different crops used in this study. Correlation between wastewater and crops were determined (Fig. S1). A Strong positive linear correlation was found in Cu (r^2^ = 0.913) followed by Zn (r^2^ = 0.804) then pb (r^2^ = 0.752), Ni (r^2^ = 0.694) and Cr (r^2^ = 0.587). These parameters showed high dependencies on each others and toxic metals in wastewater have substantial effects on crops. Yamin et al.^53^ determined the wastewater quality of Faisalabad and found that Pharang and Madhuana drains that are begin used for irrigation and generating severe health hazards in local communities. Correlation was found in soil and crops toxic metals. In Fig. S2, a strong correlation was found in Fe (r^2^ = 0.639) followed by Pb (r^2^ = 0.10) and then Ni (r^2^ = 0.08), Cr (r^2^ = 0.07) and Cu (r^2^ = 0.006). However, no negative correlation was found. These results showed the effect of soil on crops.

**Table 2:** Mean concentrations (mg kg^−1^) of potentially toxic elements in different wastewater irrigated crops.

Metals	Corn	Rice	Wheat	Sugarcane	Millet	FAO/WHO 2001 Limits* mg kg^−1^
	Mean ± SD	Mean ± SD	Mean ± SD	Mean ± SD	Mean ± SD	
Cu	2.23 ± 0.13	2.09 ± 0.11	2.25 ± 0.53	9.94 ± 1.03	10.51 ± 0.9	40
Range	2.1–2.35	1.97–2.19	1.84–2.85	9.06–11.08	9.69–11.47	
Zn	59.04 ± 6.2	55.18 ± 6.06	55.41 ± 18.7	79.63 ± 22.6	89.52 ± 17.5	60
Range	51.6–63.7	48.25–59.5	40.2–76.32	61.01–104.8	73.07–108	
Mn	25.9 ± 5.63	24.2 ± 5.25	20.2 ± 8.04	5.56 ± 2.71	6.63 ± 2.15	500
Range	21.7–32.3	20.3–30.2	14.93–29.5	3.42–8.61	4.65–8.91	
Cr	3.89 ± 0.5	3.63 ± 0.47	4.01 ± 1.40	9.86 ± 2.96	8.50 ± 1.65	2.3
Range	3.32–4.24	3.1–3.97	2.4–4.9	6.72–12.6	6.96–10.25	
Pb	2.49 ± 0.31	2.33 ± 0.29	2.97 ± 1.01	0.70 ± 0.01	0.71 ± 0.02	0.3
Range	2.31–2.85	2.16–2.67	2.15–4.1	0.7–0.71	0.7–0.73	
Fe	99.8 ± 10	93.3 ± 9.3	87.3 ± 8.81	73.3 ± 7.6	77.4 ± 6.6	–
Range	89–108.8	83.23–101.7	77.8–95.2	66.8–81.73	71.4–84.5	
Ni	2.01 ± 0.49	1.88 ± 0.45	1.54 ± 0.88	0.2 ± 0.15	0.26 ± 0.12	66.9
Range	1.63–2.56	1.52–2.39	0.97–2.55	0.09–0.37	0.15–0.38

**Table 3:** Mean concentrations (mg kg ^-1^) of potentially toxic elements in different wastewater irrigated soil.

Soil samples	Cu	Zn	Mn	Cr	Pb	Fe	Ni
Corn soil	20.8 ± 1.11	321.2 ± 35.07	21.8 ± 1.70	10.1 ± 0.34	11.3 ± 1.24	887.4 ± 89.13	20.5 ± 1.30
Range	19.6–21.8	291–360	20.1–23.5	9.82–10.5	10.29–12.7	791–967	19.17–21.7
Rice soil	19.47 ± 1.03	300.40 ± 32.92	20.40 ± 1.55	9.50 ± 0.32	10.57 ± 1.12	829.70 ± 83.30	19.14 ± 1.21
Range	18.36–20.4	272.7–336.8	18.8–21.9	9.18–9.82	9.62–11.8	739.7–904.1	17.92–20.3
Wheat Soil	22.44 ± 1.54	314.13 ± 50.28	23.23 ± 2.93	10.90 ± 0.32	12.52 ± 1.38	792.50 ± 52.91	22.53 ± 1.45
Range	21.3–24.2	260.5–360.5	20.1–25.9	10.64–11.2	11.39–14.0	740.3–846.1	21.22–24.08
Sugarcane soil	19.93 ± 0.08	305.70 ± 53.87	20.63 ± 3.16	10.18 ± 0.40	10.95 ± 0.68	756.83 ± 29.68	20.61 ± 0.33
Range	19.84–19.98	243.5–336.8	18.8–24.28	9.95–10.64	10.16–11.34	739.7–791.1	20.23–20.8
Millet soil	21.62 ± 0.21	293.73 ± 57.56	23.97 ± 3.35	10.79 ± 0.13	11.63 ± 0.42	827.77 ± 31.75	21.58 ± 0.62
Range	21.37–21.74	260.5–360.5	20.1–25.9	10.64–10.87	11.39–12.12	791.1–846.1	21.22–22.29
EU standards 2006	100	300	2,000	100	100		50

According to a study, Zn contents were found higher (33%) in wheat grains, (67%) mustard and (23%) in rice grains^54^ in a semi-arid region. Vital micronutrients including Cu and Zn are the most plentiful metals in rice and wheat^30^. Incessant elimination of toxic elements by food crops grown up in wastewater irrigated soil and metals leaching into the profounder soil layers can lower metal concentration^55^. The build-up of toxic elements in plants also depends on plant parts and its age^15^. In Egypt, a researcher had also find high values of Cd and Zn in different crops/vegetables^56^. With the fact, that toxic elements pose significant photo-toxic effects in low concentrations and therefore inhibit plant growth, that also pose serious hazards to humans through contamination of food chain^27,57^. Ingesting of toxic elements via pretentious crops can cause different diseases such as hypertension, brain damage, impair growth, lung cancer and ulcers, heart failure, low blood pressure, hepatic neurosis, skeletal abnormalities, myocardial infestation, alcopia tumour and many others^58^. Results from the present study and previous researches on South-eastern regions including Pakistan, China and India^15,27,55,57,59,60^ proven that plants grown on wastewater irrigated soils are polluted with toxic elements and pose serious health issues in local communities. Occurrence of toxic elements propose intrusion of anthropogenic influences which is accountable for contributing into the soils at approximate extent. In soil, presence of alkalinity and calcium carbonates can increase concentration of Zn^54^. The values of metals in soil were 1.11–5 times higher than background values in a study conducted by Sawut et al.^35^. Occasionally, the differences in concentrations of toxic elements can also be affected by changes in type of vegetation cover, lithological inputs, geological features, cultural influences, hydrological effects^61^.

### Pollution indices of toxic element

**Figure 2:**
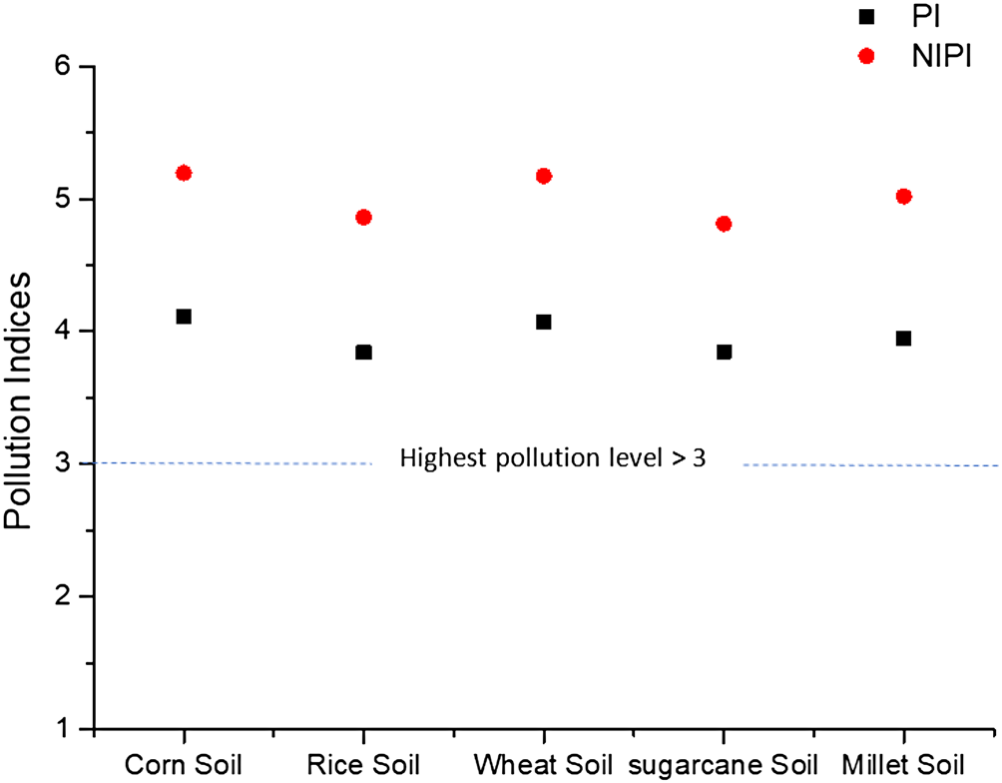
Nemerow integrated pollution index (NIPI) in soil samples.

**Figure 3:**
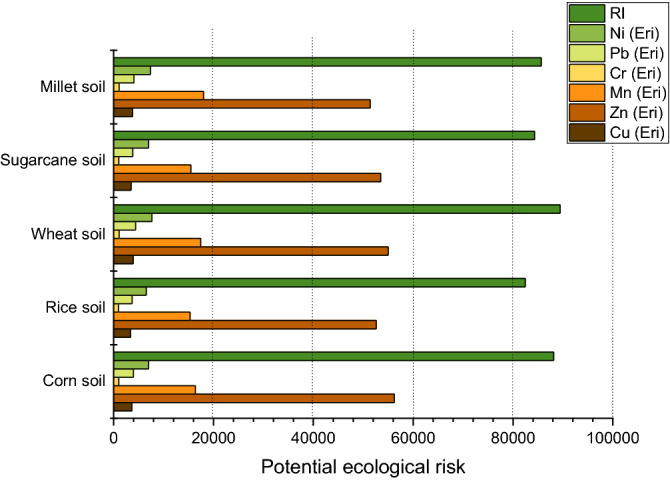
Potential ecological risk index of wastewater irrigated soil.

Nemerow integrated pollution index (NIPI) of toxic elements including Cu, Cr, Fe, Pb, Ni, Mn and Zn in soil were evaluated. For this purpose, firstly pollution index (PI) were calculated in soil samples. Results showed (Fig. 2) that all the samples were above the highest pollution limit i.e. > 3. PI values in samples were corn soil, rice soil, wheat soil, sugarcane soil and millet soil were as PI = 4.10, PI = 3.84, PI = 4.07, PI = 3.84 and PI = 3.94 respectively. Same as PI, the values of NIPI were also exceeded in all soil samples than highest level of pollution index. The order for highest to less polluted samples were observed as corn soil (NIPI = 5.19) > wheat soil (NIPI = 5.17) > millet soil (NIPI = 5.01) > rice (NIPI = 4.86) > sugarcane (NIPI = 4.81). High degrees of pollution in vegetable based soil is may due to irrational agricultural activities like Unnecessary use of pesticides, synthetic fertilizers and consequence of large industries that may be situated adjacent to these cultivation bases^62^. PERI (Potential ecological pollution index) of multiple toxic metals were calculated. Results indicated that all metals (Cr, Cu, Pb, Mn, Ni and Zn) were more than the heavy ecological pollution risk i.e. E_r_^i^ > 320. However, the highest E_r_^i^ was found in zinc in all soil samples. The order of highest to lowest was as follow: Zn > Mn > Ni > Pb > Cu > Cr. The risk index (RI) was calculated which found higher in all samples than the heavy ecological pollution risk index i.e. PERI > 600. The order was following: Wheat soil (PERI = 89,454) > Corn soil (PERI = 88,135) > Millet soil (PERI = 85,650) > Sugarcane soil (PERI = 84,315) > Rice soil (PERI = 82,434) as shown in Fig. 3. The results of Ecological risk assessment and Nemerow pollution index were when related with numerous researches^35,63,64^ they found higher in present study. Polluted soil can be effectually use for timber production, cultivation of ornamental plants, and construction material as a substitute of agronomic crops to decrease the ecological and environmental risk^65^.

### Bioconcentration factor (BCF)

**Figure 4:**
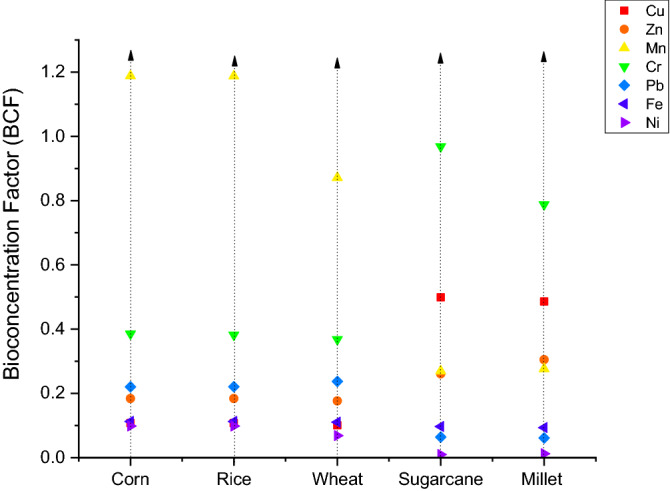
Bioconcentration factor for crop grown in wastewater.

Figure 4 shown the values of bioconcentration factor from soil to crop samples. Manganese had the highest BCF value among all elements. The maximum BCF in Mn was found in Corn soil (1.188) followed by Rice soil (1.187). The BCF values was ordered as: Mn > Zn > Cr > Pb > Fe > Cu > Ni. Among all the crop samples Corn exhibit the highest BCF value followed by Rice and then sugarcane. The complete order was as: Corn > Rice > Sugarcane > Millet > Wheat. Toxic elements that have more value of bioconcentration factor, have more chances and easier way of accumulating in plants/crops and translocate to edible parts than toxic elements with low bioconcentration factor and can cause more health risks ^20,66^. These toxic elements pledge and develop the neoplastic course by triggering DNA alterations and by release of oxygen free radicals ^67^. Parallel BCF results were detected from earlier researches in different areas (including Sialkot, Gujranwala, and Swat) of Pakistan. These results were protuberant in terms of long-term wastewater irrigation in which the same properties in the soil would not be the part of food chain^27^.

### Predicted daily intake exposure of toxic elements

Mean concentrations of toxic metals were taken to evaluation of Daily intake exposure (DIE) in adult and children via crop consumption (mg kg^−1^ day^−1^). The daily intake exposure values were shown in Table S4. Results showed (Table 4) from health risk index that Cu (1.22E+00 and 1.29E+00), Zn (1.31E+00 and 1.47E+00) and Cr (1.39E+00 and 1.20E+00) in sugarcane and millet respectively, Mn (3.87E+00, 3.62E+00 and 3.02E+00) and Pb (3.07E+00, 2.87E+00 and 3.66E+00) in corn, rice and wheat, respectively was near threshold level (HRI > 1) in adults. In children, the HRI values in Cu (1.76E+00 and 1.86E+00) and Cr (2.00E+00 and 1.72E+00) was near threshold level in sugarcane and millet respectively, while in Zn (1.40E+00, 1.30E+00, 1.31E+00, 1.88E+00 and 2.12E+00), Mn (5.57E+00, 5.21E+00, 4.35E+00, 1.20E+00 and 1.43E+00) and Pb (4.42E+00, 4.13E+00, 5.27E+00, 1.24E+00 and 1.26E+00) it was higher in all crop samples including corn, rice, wheat, sugarcane and millet respectively. Nickel was found lower than the permissible limit HRI > 1 in adults and children in all crop samples. There is a level of toxicity present in crop consumption as described by USEPA^46^ and this toxicity could be increase via consumption of these wastewater irrigated crops.

**Table 4:** Health risk index (HRI) Hazard Quotient (HQ) for non-carcinogenic and carcinogenic risk assessment via crop consumption.

Group	Health risk assessment (HRI)							Cancer risk	
	Crops	Cu	Zn	Mn	Cr	Pb	Ni	Cr	Pb
Adult	Corn	2.75E−01	9.70E−01	3.87E+00	5.48E−01	3.07E+00	4.95E−01	4.11E−03	4.47E−05
	Rice	2.58E−01	9.07E−01	3.62E+00	5.11E−01	2.87E+00	4.63E−01	3.83E−03	4.18E−05
	Wheat	2.77E−01	9.10E−01	3.02E+00	5.65E−01	3.66E+00	3.80E−01	4.24E−03	5.33E−05
	Sugarcane	1.22E+00	1.31E+00	8.30E−01	1.39E+00	8.63E−01	4.93E−02	1.04E−02	1.26E−05
	Millet	1.29E+00	1.47E+00	9.90E−01	1.20E+00	8.75E−01	6.41E−02	8.98E−03	1.27E−05
Children	Corn	3.96E−01	1.40E+00	5.57E+00	7.89E−01	4.42E+00	7.13E−01	1.18E−03	1.29E−05
	Rice	3.71E−01	1.30E+00	5.21E+00	7.36E−01	4.13E+00	6.67E−01	1.10E−03	1.20E−05
	Wheat	3.99E−01	1.31E+00	4.35E+00	8.13E−01	5.27E+00	5.46E−01	1.22E−03	1.54E−05
	Sugarcane	1.76E+00	1.88E+00	1.20E+00	2.00E+00	1.24E+00	7.09E−02	3.00E−03	3.62E−06
	Millet	1.86E+00	2.12E+00	1.43E+00	1.72E+00	1.26E+00	9.22E−02	2.58E−03	3.67E−06

Group	Targeted hazard quotient (THQ)							Hazard index (HI)	
	Crops	Cu	Zn	Mn	Cr	Pb	Ni		
Adult	Corn	2.75E−01	9.70E−01	3.87E+00	5.48E−01	3.07E+00	4.95E−01	9.22E+00	
	Rice	2.58E−01	9.07E−01	3.62E+00	5.11E−01	2.87E+00	4.63E−01	8.63E+00	
	Wheat	2.77E−01	9.10E−01	3.02E+00	5.65E−01	3.66E+00	3.80E−01	8.82E+00	
	Sugarcane	1.22E+00	1.31E+00	8.30E−01	1.39E+00	8.63E−01	4.93E−02	5.66E+00	
	Millet	1.29E+00	1.47E+00	9.90E−01	1.20E+00	8.75E−01	6.41E−02	5.89E+00	
Children	Corn	3.96E−01	1.40E+00	5.57E+00	7.89E−01	4.42E+00	7.13E−01	1.33E+01	
	Rice	3.71E−01	1.30E+00	5.21E+00	7.36E−01	4.13E+00	6.67E−01	1.24E+01	
	Wheat	3.99E−01	1.31E+00	4.35E+00	8.13E−01	5.27E+00	5.46E−01	1.27E+01	
	Sugarcane	1.76E+00	1.88E+00	1.20E+00	2.00E+00	1.24E+00	7.09E−02	8.15E+00	
	Millet	1.86E+00	2.12E+00	1.43E+00	1.72E+00	1.26E+00	9.22E−02	8.48E+00	

### Non-carcinogenic and carcinogenic risk assessment

In developing countries like Pakistan, health risk assessment is very important due to frequent usage of untreated wastewater as irrigation for crop yields^20^. Targeted hazard quotient (THQ) for non-carcinogenic risk assessment was calculated in adult and children for all crop samples (Table 4). THQ values in Cu, Zn and Cr was found near threshold level in sugarcane and millet while Mn and Pb were found near threshold level in corn, rice and wheat in adults. In children, Cu and Cr in sugarcane and millet while in Zn, Mn and Pb in all crop samples were near THQ 1. The sum of THQs were calculated in form of Hazard Index (HI), its values in adult were near threshold level (HI > 1) in all samples (Corn = 9.22E+00, Rice = 8.63E+00, Wheat = 8.82E+00, Sugarcane = 5.66E+00 and Millet = 5.89E+00). In children the HI values in Corn (1.33E+01), Rice (1.24E+01) and Wheat (1.27E+01) were above threshold limit while in Sugarcane (8.15E+00) and Millet (8.48E+00) were near threshold level. It was clear from the results that children pose more non-carcinogenic risk than adult via consumption of all these crops. Similar study was reported by Xiao et al.^68^.

For carcinogenic risk, Cr and Pb were evaluated based on available slope factors. Table 4 showed the cancer risk (CR) in Pb was found slightly higher than the safe level (1 × 10^–6^) as described by USEPA^48^ in all crop samples in adult (Corn = 4.47E−05, Rice = 4.18E−05, Wheat = 5.33E−05, Sugarcane = 1.26E−05 and Millet = 1.27E−05) and in children it was higher in corn (1.29E−05), rice (1.20E−05) and wheat (1.54E−05). The CR values for Cr was found very high both in adult and children in all crop samples. The CR values in Cr were as follow: Corn = 4.11E−03, Rice = 3.83E−03, Wheat = 4.24E−03, Sugarcane = 1.04E−02 and Millet = 8.98E−03 in adults, while in children Corn = 1.18E−03, Rice = 1.10E−03, Wheat = 1.22E−03, Sugarcane = 3.00E−03 and Millet = 2.58E−03. Cr as seemed to be predominant contaminant and the main source that create a relatively higher cancer risk outside the acceptable limit as compared to Pb. The result for Cr in this study was more than stated by Yousaf et al.^26^ in different food samples both in adult and children. However, there are further exposure pathways via inhalation to oral, dermal intake which require further study. Urbanization strongly require the remediation strategies for urban and peri-urban soil to reduce the risk of metallic elemental exposure in local communities^16,26,69^. Pollution through anthropogenic sources have different characteristics containing multi environmental impacts with high number of toxic elements^70^.

## Conclusion

This study has elaborated the effects of agricultural utility of untreated wastewater on soil and crops. High level of pollution in wastewater leads higher contamination of soil and crops. The determined concentration of pollutants and proposed risk assessment are significantly higher than the permissible limits by FAO and WHO. Whereas, such exceeding levels of pollution parameters pose potential health hazards in humans and there are many reported health-related problems like kidney and liver diseases mainly, being caused due to chromium ingestion. While, Government and other agencies are aware about this practice but presently there are policy and legation gaps regarding treatment and safe reuse of such wastewater. Therefore, the Governments and industries should take collective responsibilities to treat such wastewater streams prior to disposal and create awareness in farmers for their effective and right reuse for right cropping systems. The study will be therefore be quite supportive for the policy makers and other stakeholders for the provision of basic data, information and awareness so to minimize the risk of exposure, environmental sustainability, and raising the food security issues for local communities.

## Supplementary information

Supplementary Information 1.
