# Supplementary material for: Critical risk analysis of metals toxicity in wastewater irrigated soil and crops: a study of a semi-arid developing region
Source: Sci Rep. 2020 Jul 30;10:12845. doi: 10.1038/s41598-020-69815-0 (PMC7393170; doi:10.1038/s41598-020-69815-0)
Supplement: Supplementary file 1 — Supplementary Information 1. [file 41598_2020_69815_MOESM1_ESM.docx]

**Critical risk analysis of metals toxicity in wastewater irrigated soil and crops – a study of a semi-arid developing region**

Yusra Mahfooz^1^, Abdullah Yasar^1^, Liu Guijian^2^, Qamer Ul Islam^3,4^, Amtul Bari Tabinda Akhtar^1^, Rizwan Rasheed^1,5^, Samina Irshad^2^, Urooj Naeem^1^

*^1^Sustainable Development Study Centre, Government College University, Lahore, Punjab 54000, Pakistan*

*^2^Chinese Academy of Science (CAS)-Key Laboratory of Crust-Mantle Materials and the Environments, School of Earth and Space Sciences, University of Science and Technology of China, Hefei 230026, Anhui, People’s Republic of China*

*^3^District Officer Planning, City District Government Gujranwala, Pakistan*

*4Department of Architecture and Town Planning, University of Engineering and Technology*

*Lahore*

*^5^Department of Architecture and Built Environment, University of Nottingham, Nottingham NG7 2RD, UK*

**Corresponding Author:**

Yusra Mahfooz

*^1^Sustainable Development Study Centre, Government College University, Lahore, Punjab 54000, Pakistan*

[sdsc8888@gmail.com](mailto:sdsc8888@gmail.com)

**Table S1: Categories for NIPI and ecological risk levels**

| **Pollution index (PI)** | **Nemerow integrated pollution index (NIPI)** | **Pollution level** |
| --- | --- | --- |
|  | < 0.7 | No pollution |
| PI < 1 | 0.7 > NIPI < 1 | Threshold line of pollution |
| 1 > PI < 2 | 1 > NIPI < 2 | Low pollution |
| 2 > PI < 3 | 2 > NIPI < 3 | Moderate pollution |
| PI > 3 | NIPI > 3 | High pollution |
| **Potential ecological risk index (RI)** | **Potential risk coefficient (E_r_^i^)** | **Risk level** |
| RI < 150 | < 40 | Low |
| 150 ≤ RI < 300 | 40 < E_r_^i^ > 80 | Moderate |
| 300 ≤ RI < 600 | 80 < E_r_^i^ > 160 | High |
| RI ≤ 600 | 160 < E_r_^i^ > 320 | Slightly heavy |
| RI > 600 | E_r_^i^ > 320 | heavy |

**Table S2: Detail of selected crops**

| **Sr #** | **Botanical name** | **Common name** | **Edible part** | **Plant Height (cm)** |
| --- | --- | --- | --- | --- |
| 1 | *Saccharum officinarum* | Sugarcane | Stem | 365 |
| 2 | *Zea mays* | Corn | Corn | 198 |
| 3 | Triticum aestivum | Wheat | Grains | 106 |
| 4 | *Oryza sativa* | Rice | Caryopsis | 121 |
| 5 | *Pennisetum glaucum* | Millet | Grains | 172 |

**Table S3: Physical properties of wastewater irrigated soil**

| **Parameters** | **Unit** | **CS** | **RS** | **WS** | **SS** | **MS** |
| --- | --- | --- | --- | --- | --- | --- |
| Sand (%) | (>20µm) | 44.49±2.18 | 46.58±2.09 | 43.69±1.97 | 45.83±2.71 | 42.67±1.97 |
| Silt (%) | (2µm-20 µm) | 28.36±1.11 | 24.13±1.13 | 28.84±1.56 | 27.42±2.11 | 27.38±2.13 |
| Clay (%) | (<2µm) | 27.14±1.21 | 29.28±0.98 | 27.46±0.96 | 26.74±1.17 | 29.94±1.13 |
| CEC* | cmolkg^-1^ | 11.56±1.18 | 13.12±1.31 | 10.78±0.34 | 11.23±1.12 | 12.34±1.07 |
| pH | -- | 7.73±0.96 | 7.49±0.32 | 8.12±0.89 | 7.63±0.96 | 7.45±1.09 |
| EC** | dS m^-1^ | 2.45±0.48 | 3.62±0.61 | 2.13±0.64 | 1.99±0.69 | 2.94±1.07 |
| OM*** | % | 1.23±0.42 | 1.45±0.37 | 1.18±0.82 | 1.09±0.21 | 1.26±0.55 |

N=4, SS= Sugarcane soil, CS= Corn soil, WS= Wheat soil, RS= Rice soil, MS= Millet soil

*Cation Exchange Capacity

**Electric Conductivity

***Organic Matter

Soil texture in all samples fall under Loam clayey- loam class similar with Yousaf et al., (2016).

**Table S4:** Daily Intake Exposure (DIE) of metals via crop consumption in human

|  | **Crops** | **Cu** | **Zn** | **Mn** | **Cr** | **Pb** | **Fe** | **Ni** |
| --- | --- | --- | --- | --- | --- | --- | --- | --- |
| **Adult** | Corn | 9.34E-04 | 2.47E-02 | 1.09E-02 | 1.63E-03 | 1.04E-03 | 4.18E-02 | 8.42E-04 |
|  | Rice | 8.76E-04 | 2.31E-02 | 1.02E-02 | 1.52E-03 | 9.76E-04 | 3.91E-02 | 7.88E-04 |
|  | Wheat | 9.43E-04 | 2.32E-02 | 8.48E-03 | 1.68E-03 | 1.24E-03 | 3.66E-02 | 6.45E-04 |
|  | sugarcane | 4.16E-03 | 3.34E-02 | 2.33E-03 | 4.13E-03 | 2.93E-04 | 3.07E-02 | 8.38E-05 |
|  | Millet | 4.40E-03 | 3.75E-02 | 2.78E-03 | 3.56E-03 | 2.97E-04 | 3.24E-02 | 1.09E-04 |
| **Children** | Corn | 1.34E-03 | 3.56E-02 | 1.56E-02 | 2.35E-03 | 1.50E-03 | 6.02E-02 | 1.21E-03 |
|  | Rice | 1.26E-03 | 3.33E-02 | 1.46E-02 | 2.19E-03 | 1.41E-03 | 5.63E-02 | 1.13E-03 |
|  | Wheat | 1.36E-03 | 3.34E-02 | 1.22E-02 | 2.42E-03 | 1.79E-03 | 5.27E-02 | 9.29E-04 |
|  | sugarcane | 5.99E-03 | 4.80E-02 | 3.35E-03 | 5.95E-03 | 4.22E-04 | 4.42E-02 | 1.21E-04 |
|  | Millet | 6.34E-03 | 5.40E-02 | 4.00E-03 | 5.13E-03 | 4.28E-04 | 4.67E-02 | 1.57E-04 |

**Soil risk assessment**

Hazard index was calculated for soil samples through different pathways. The formulas for calculating Chronic daily Intake (CDI) for ingestion and dermal are as follow:

CDI _ingest‐soil_ = $\frac{Cs\times IRS\times EF\times ED}{BW\times AT}xCF$ (Eq. S1)

CDI _Derm‐soil_ = $\frac{Cs\times SA\times AF\times ABS\times ED}{BW\times AT}xCF$ (Eq. S2)

Here, Cs indicate concentration of potential metal in sample, IRS is ingestion rate, EF is exposure frequency, ED is exposure duration, SA is skin area exposed, AF is adherence factor, ABS is dermal absorption fraction (USEPA, 2011), BW is body weight (USEPA, 1991) and AT is average time for non-carcinogen and carcinogen (USEPA, 2011, 2002).

Hazard Quotient was calculated for non-carcinogenic risk by using following formula:

HQ = CDI / RfD (Eq. S3)

HI = $\sum_{i=1}^{n} \mathrm{HQ}$ (Eq. S4)

RfD is the chronic reference dose (mg/kg/day). The values for RfD for ingestion and dermal were taken from literature for each metal (Li et al., 2015; USEPA 2014; Liang et al., 2017; Lu et al., 2014; Chen et al., 2015).

For carcinogenic risk assessment, the following formula was taken

Cancer Risk = CDI x SF (Eq. S5)

Where CDI is chronic daily intake and SF is cancer slope factor (mg/kg/day) (Liang et al., 2017; Lim et al., 2008).

**Result**

The maximum hazard index was found via ingestion than dermal pathway of all soil samples. Non carcinogenic risk started when Hazard quotient increases than 1 (HQ >1) (Mahfooz et al., 2019). The carcinogenic risk assessment was found more in Cr in all samples (2.49E-04 in corn soil, 2.34E-04 in rice soil, 2.69E-04 in wheat soil, 2.51E-04 in sugarcane soil and 2.66E-04 in millet soil) than Ni. However, the carcinogenic hazard found at between 1 × 10^−4^ to 1 × 10^−6^. So, all the samples were found at potential hazard stage (Table S4).

**Table S5:** Health Risk Index and carcinogenic risk assessment in soil

| **Soil** | **Hazard Index (HI)** | | **Cancer Risk (CR)** | |
| --- | --- | --- | --- | --- |
|  | **HI_ing_** | **HI_derm_** | **Cr** | **Ni** |
| Corn soil | 1.35E-02 | 1.10E-03 | 2.49E-04 | 1.01E-05 |
| Rice soil | 1.27E-02 | 1.04E-03 | 2.34E-04 | 9.43E-06 |
| Wheat soil | 1.46E-02 | 1.19E-03 | 2.69E-04 | 1.11E-05 |
| Sugarcane soil | 1.33E-02 | 1.10E-03 | 2.51E-04 | 1.01E-05 |
| Millet soil | 1.40E-02 | 1.17E-03 | 2.66E-04 | 1.06E-05 |

**References**

Chen, H.; Teng, Y.; Lu, S.; Wang, Y.; Wang, J. (2015), Contamination features and health risk of soil heavy metals in China. Sci. Total Environ. 512–513, 143–153.

Li P., Lin C., Cheng H., Duan X., Lei K., 2015. Contamination and health risks of soil heavy metals around a lead/zinc smelter in southwestern China, Ecotoxicology and Environmental Safety 113, 391–399.

Liang Y., Yi X., Dang Z., Wang Q., Luo H., Tang J., 2017. Heavy Metal Contamination and Health Risk Assessment in the Vicinity of a Tailing Pond in Guangdong, China, Int. J. Environ. Res. Public Health 14, 1557. doi:10.3390/ijerph14121557

Lim, H.S., Lee, J.S., Chon, H.T., Sager, M., 2008. Heavy metal contamination and health risk assessment in the vicinity of the abandoned Songcheon Au-Ag mine in Korea. J. Geochem. Explor. 96, 223–230.

Lu, X., Zhang, X., Li, L.Y., Chen, H., 2014. Assessment of metals pollution and health risk in dust from nursery schools in Xi’an, China. Environ. Res. 128, 27–34.

Mahfooz, Y., Yasar, A., Sohail, M. T., Tabinda, A. B., Rasheed, R., Irshad, S., Yousaf, B., 2019. Investigating the drinking and surface water quality and associated health risks in a semi-arid multi-industrial metropolis (Faisalabad), Pakistan, Environmental Science and Pollution Research 26, 20853–20865.

USEPA (United States Environmental Protection Agency), 2014. Integrated risk information system (IRIS), 〈http://www.epa.gov/iris/〉.

|  |  |
| --- | --- |
|  |  |
|  |  |

Figure S1: Correlation coefficient of PTEs between wastewater and crop samples

|  |  |
| --- | --- |
|  |  |
|  |  |

Figure S2: Correlation coefficient of PTEs between soil and crop samples
